# Supplementary material for: Nanoarchitectonics of Electrically Activable Phosphonium Self-Assembled Monolayers to Efficiently Kill and Tackle Bacterial Infections on Demand
Source: Int J Mol Sci. 2022 Feb 16;23(4):2183. doi: 10.3390/ijms23042183 (PMC8879818; doi:10.3390/ijms23042183)
Supplement: Supplementary file 1 [file ijms-23-02183-s001.zip › ijms-1582113-supplementary.pdf]

Supporting Information

Development of electrically activable phosphonium self-assembled monolayers to efficiently kill and tackle bacterial infections on-demand.

Serena Carrara,[1#†] Florent Rouvier,[2†] Sanjana Auditto,[1] Frédéric Brunel,[1] Charlotte Jeanneau,[3] Michel Camplo,[1] Michelle Sergent,[4] Imad About,[3] Jean-Michel Bolla[2*] and Jean-Manuel Raimundo[1*]

[1] Aix-Marseille Univ, CNRS, CINAM, Marseille, France.

[2] Aix-Marseille Univ, INSERM, SSA, IRBA, MCT, Marseille, France.

[3] Aix-Marseille Univ, CNRS, ISM, Inst Movement Sci, Marseille, France.

[4] Aix-Marseille Université, Avignon Université, CNRS, IRD, IMBE, Marseille, France.

Table of Contents

1.1 Synthesis 2

1.2 Design of Experiments 3

1.1 Synthesis

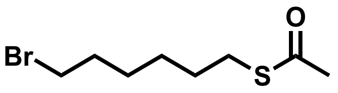

S-(6-Bromohexyl) ethanethioate. (1). Dibromohexane (6 g; 24.6 mmol; 5 eq.) into acetone (125 mL) was added potassium thioacetate (561 mg; 5 mmol; 1 eq.). Then the mixture was stirred at room temperature for 16 hours. The solvent was then evaporated under high vacuum and the obtained residue was taken up into water (75 mL), the aqueous phase was extracted with DCM (3 x 50 mL). The combined organic layers were washed with brine (50 mL), dried over $MgSO_4$, filtered and evaporated under vacuum. The product was purified over SiO_2 column flash chromatography (EP / AcOEt ; 95 : 5) to give a colorless oil (779 mg ; 3.26 mmol ; 65% yield). ^1H NMR (400 MHz ; $CDCl_3$): δ 3.39 (t, 2H, *J* 6.6 Hz), 2.85 (t, 2H, *J* 7.4 Hz), 2.31 (s, 3H), 1.84 (quint, 2H, *J* 7.2 Hz), 1.57 (quint, 2H, *J* 7.2 Hz), 1.41 (m, 4H). ^{13}C (101 MHz, $CDCl_3$): δ 196.6, 33.1, 32.6, 30.7, 29.4, 29, 27.9, 27.4. ESI MS : 239.1 m/z : $[M+H]^+$; 245.0 m/z : $[M+Li]^+$.

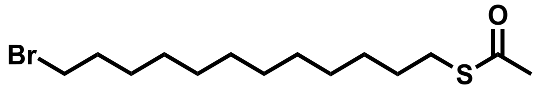

S-(6-Bromododecyl) ethanethioate (2). To a solution of 1,12-dibromododecane (5 g; 15.2 mmol; 5 eq.) into acetone (125 mL) was added potassium thioacetate (348 mg; 3 mmol; 1 eq.) under inert atmosphere. Then the mixture was let to react at room temperature for 16 hours. The solvent was then evaporated under high vacuum and the obtained residue was taken up into water (150 mL) and the aqueous phase was extracted with DCM (3 x 100 mL). The combined organic layers were washed with brine (100 mL), dried over $MgSO_4$, filtered and evaporated under vacuum. The product was purified over SiO_2 column flash chromatography (EP : AcOEt ; 95 : 5) to give a colorless oil (721 mg ; 2.23 mmol; 74% yield). ^1H NMR (400 MHz ; $CDCl_3$): δ 3.39 (t, 2H, *J* 6.8 Hz), 2.85 (t, 2H, *J* 7.4 Hz), 2.31 (s, 3H), 1.84 (quint, 2H, *J* 7 Hz), 1.53 (quint, 2H, *J* 7.2 Hz), 1.40 (quint, 2H, *J* 7.2 Hz), 1.25 (br, 14H). ^{13}C NMR (101 MHz ; $CDCl_3$): δ 196.2, 34.2, 32.9, 30.7, 29.6, 29.2, 28.9, 28.2 ; 6 signals obscured or overlapping. ESI MS : 325 m/z : $[M+H]^+$; 342.1 m/z : $[M+NH_4]^+$; 346.9 m/z : $[M+Na]^+$.

1.2 Design of Experiments

	Number of levels	Name /Value
Factor 1	3	Contact time
Level 0		30 min
Level 1		60 min
Level 2		120 min
Factor 2		Voltage
Level 0	4	CA 0.2
Level 1		CA 0.5
Level 2		CA 0.8
Level 3		CVM
Factor 3	7	Ionic Liquid
Level 0		Without
Level 1		6-12 50%
Level 2		6-12 100%
Level 3		8-6 50%
Level 4		8-6 100%
Level 5		8-12 50%
Level 6		8-12 100%

Figure S1. Factors and levels for DOE matrix preparation.

Experiment	Ionic Liquid	Contact time	Voltage
1	Without	30	CA 0.2V
12	Without	120	CVM
20	6-12 50%	60	CA 0.8V
22	6-12 50%	30	CVM
29	6-12 100%	60	CA 0.5V
36	6-12 100%	120	CVM
42	8-6 50%	120	CA 0.5V
43	8-6 50%	30	CA 0.8V
50	8-6 100%	60	CA 0.2V
52	8-6 100%	30	CA 0.5V
57	8-6 100%	120	CA 0.8V
66	8-12 50%	120	CA 0.5V
71	8-12 50%	60	CVM
75	8-12 100%	120	CA 0.2V
79	8-12 100%	30	CA 0.8V
83	8-12 100%	60	CVM

Figure S2. Critical factors extrapolated from the complex matrix for DOE.
